# Comprehensive Sequence Analysis of *IQD* Gene Family and their Expression Profiling in Grapevine (*Vitis vinifera*)

**DOI:** 10.3390/genes11020235

**Published:** 2020-02-24

**Authors:** Zhongjie Liu, Muhammad Salman Haider, Nadeem Khan, Jinggui Fang

**Affiliations:** 1 Key Laboratory of Genetics and Fruit Development, College of Horticulture, Nanjing Agricultural University, Nanjing 210095, China; 2017204011@njau.edu.cn (Z.L.); salman.hort1@gmail.com (M.S.H.); 2 Ottawa Research and Development Center, Agriculture and Agri-Food Canada, 960 Carling Avenue, Ottawa, ON K1A 0C6, Canada; 2016104235@njau.edu.cn; 3 Department of Biology, University of Ottawa, 30 Marie Curie, Ottawa, ON K1N 6N5, Canada

**Keywords:** grapevine, genome-wide analysis, *IQD* genes, subcellular localization, expression analysis

## Abstract

The plant-specific IQ67-domain (IQD) protein family members are downstream targets of calcium sensors, known to regulate plant growth and lateral organ polarity, and basal defense response against environmental cues. No systematic study of *IQD* gene family has been performed on grapevine. The public availability of grapevine genome enables us to perform identification, phylogeny, chromosomal orientation, and gene structure analysis of the *IQD* genes in grapevine. We identified 49 *VvIQD* genes (*VvIQD1*–*VvIQD49*) and further classified them into eight subgroups based on phylogenetic relationships. The 49 *VvIQD* genes were assigned to 19 different chromosomal positions. The collinear relationship between grapevine and *Arabidopsis* IQDs (*VvIQD* and *AtIQD*), and within grapevine *VvIQDs*, was highly conserved. In addition, most of duplicated gene pairs showed *Ka*/*Ks* ratio less than 1.00, indicating purifying selection within these gene pairs, implying functional discrepancy after duplication. Transcription profiling of *VvIQD* genes shed light on their specific role in grapevine tissue and organ development. The qRT-PCR validation of the 49 *VvIQD* genes in grape berry tissue from cultivars with distinct berry shape during developmental phases suggested candidate genes involved in the shape of grape berries. The subcellular prediction of *VvIQD22*, *VvIQD23*, *VvIQD38*, and *VvIQD49* genes validated their localization in the nucleus and plasma membrane. The VvIQD49 protein interaction with *VvCaM2* was also verified by bimolecular fluorescence complementation (BiFC) analysis in the plasma membrane. Our findings will be valuable for the functional genomic studies for desirable shape development of grape berries.

## 1. Introduction

Harvestable plant organs exhibit significant phenotypic diversity between and within species. This diversity is not only vital for marketing produce (fruits, flowers, vegetables, seeds, tubers, and leaves) but also provides the basis for selection of useful agronomic traits (size and shape) [1]. Recent research demonstrated the role of genes that interact with other proteins and link with microtubules, altering cell division patterns that control the specific form of vegetative or reproductive organs [2,3]. The supracellular alignment of microtubule array dynamics, maximal tensile strength, control cellulose microfibrils deposition, and both cell division and expansion, lead to the development of distinct organ shape and size during growth [4,5].

Various proteins (i.e., G-protein signaling, transcription factors, UBI-proteasome pathway) and phytohormones (i.e., auxin, cytokinin, and brassinolide) control the shape of harvestable organs [6,7]. In tomato, the OFP class gene (*OVATE*) and *SlOPF20* interact with microtubules via Tonneaul Recruitment Motif (TRM), and mutations in both genes lead to pear shape tomato fruits [3,5]. Similarly, the IQ67 Domain (*IQD*) gene family is known to interact with calmodulin (CaM) and microtubule proteins (SPR2 and KLCR1), regulating microtubule structure [8,9]. A SUN member, which shares similar homology with *Arabidopsis AtIQD25*-*27,* has been shown to control the fruit shape of watermelon and cucumber [10,11]. To date, various *IQD* genes have been shown to control morphology, such as *AtIQD11* and *AtIQD16* in elongated shape, *AtIQD14* in twisted shape, and *AtIQD25* in round shape, demonstrating the functional diversity of IQD proteins in the regulation of cellular elongation and the cytoskeleton [8,12,13].

Grapevine is one of the earliest domesticated fruit crops (5000–8000 years ago), contributing to the social and cultural well-being of farmers [14]. According to FAO statistics, grapevine covers 7.6 million hectares with 72 million tons of grape berry production globally [15,16]. Human selection for desirable berry traits, such as color, size, shape, sweetness, acidity, etc., led to different breeding targets for table (fresh) and wine grapes [17,18]. Grape berries vary in shape; including: round, oblong, obovate, elongate, etc., which mainly depend upon the rate, pattern and duration of cell division, and the direction of cell enlargement [8]. The specific role of *IQD* gene family in determining fruit shape by altering cell division and cytoskeleton has been studied in many crops, but no study has been carried out on grape berry shape development. Extensive genome-wide analysis in grapevine was carried out, including identification, phylogenetic analysis, motif and gene structure analysis, GO and KEGG enrichment analysis, and subcellular prediction of *IQD* genes in nucleus using a tobacco system. A qRT-PCR analysis was done to determine the shape-specific candidate genes in grapevine. This study will contribute to the understanding of the underlying molecular mechanism of shape-specific candidate genes for molecular breeding of grapevine cultivars.

## 2. Materials and Methods

### 2.1. Sequence Retrieval for VvIQD Genes

For sequence retrieval and identification of IQD proteins, we used available genome databases for grapevine (Version 2.0) and its annotation http://genomes.cribi.unipd.it/grape/ and *Arabidopsis thaliana* (TAIR10: http://www.arabidopsis.org/) [19]. The sequence of other species including, *Physcomitrella patens*, *Oryza sativa*, *Solanum lycopersicum*, and *Brachypodium distachyon* were downloaded from phytozome (Version 12.1 https://phytozome.jgi.doe.gov/pz/portal.html). Afterwards, the identified proteins sequences were further verified for IQD domain compositions in SMART databases (Version 8.0) (http://smart.embl-heidelberg.de/) [20] and NCBI-Conserved Domain database (Version 3.12) (https://www.ncbi.nlm.nih.gov/Structure/cdd/wrpsb.cgi) [21]. The protein sequences with errors, shorter length (<100 bp), and absence of IQD domain were eliminated.

### 2.2. Multiple Sequence Alignment (MSA) and Phylogenetic Characterization of VvIQD Genes

To comprehend the relationship of IQDs between *Vitis vinifera* L., *A. thaliana*, *O. sativa*, *P. patens*, *S. lycopersicum*, and *B. distachyon*, the amino acid sequences of *VvIQD, AtIQD, OsIQDm PpIQD, SlIQD*, and *BdIQD* were aligned by MUSCLE and then were used to construct a phylogenetic tree using the maximum likelihood (ML) method in MEGA (Version 7.0) software with the default options [22]. The circular tree was constructed using the 1000 bootstrap replicated by the Jones, Taylor, and Thornton amino acid substitution model (JTT model), keeping the other parameters set to the defaults to determine the reliability of the resulting tree. To understand the gene duplication types, the *Ka*/*Ks* values of duplicated gene pairs were calculated using MEGA.

### 2.3. Conserved Motifs, Exon-Intron Structure Analysis, Physicochemical Parameters, and Ka/Ks for Duplicated VvIQD Genes

Multiple Em for Motif Elicitation (Version 5.0.3) were used for conserved motif scanning of *VvIQD* proteins. The parameters settings were: maximum number of motifs, 10; minimum width of 100; and a maximum width of 120, with all other parameters set to the defaults [23]. We used *V. vinifera* GFF3 file to visualize gene structure using TBtools (Version 0.66) [24]. The ExPASY PROTPARAM tool (http://web.expasy.org/protparam/) [25] was consulted for various physicochemical properties, such as molecular weight (MW), isoelectronic points (pI), aliphatic index, and GRAVY values for each gene. The subcellular localization was predicted using the WOLF PSORT (https://wolfpsort.hgc.jp/) website [22].

### 2.4. GO and KEGG Enrichment Analysis of IQD Genes in Grapevine

For GO enrichment annotations, online Panther server (http://pantherdb.org/) was used to acquire functional annotations. The online KEGG database (https://www.genome.jp/kegg/pathway.html) was consulted to deduce the critical role of *VvIQD*s in biological pathways. The illustration of GO and enriched KEGG pathways were drawn with TBtools software [26]. These analyses were largely based on providing biological insight to the IQD members in grapevine.

### 2.5. Chromosomal Location and Synteny Correlation Analysis

For chromosomal locations of *VvIQD* genes, a Circos diagram was illustrated by annotating genes on their specific chromosomal position in the genome annotation by using the TBtools software [26]. The syntenic gene relationship between the homologs of *A. thaliana* and *V. vinifera,* and within the *V. vinifera,* was also verified. These syntentic or collinear analysis were carried out by using the MCScanX with gene duplication parameters [27].

### 2.6. Plant Material and Source

Different shapes of grape berries, including: round (cv. Bashi), oblate circle (cv. Queenora seedless), nearly round (cv. 11–43), oval (cv. Guifeimeigui), and long oval (cv. Zaomanao) were collected at four different developmental stages (i.e., 20 days after flowering (DAF), 50 DAF, 80 DAF, and 110 DAF, respectively) at the National Grape Germplasm Repository at Zhengzhou Fruit Research Institute of the Chinese Academy of Agricultural Sciences (113°42′ E and 34°42′ N) during December 2018. The annual precipitation of Zhengzhou was 689.1 mm and the average annual temperature was 16 °C.

### 2.7. RNA Isolation and Expression Profiling of *V. vinifera* Fruits of Different Shapes During Their Developmental Phase

The RNA was extracted from different shapes of grape berries at their different maturity phases by using the Trizol (Invitrogen) method following the steps as described in instructions. The cDNA was prepared using the Primer Script RT reagent kit (TAKARA, Dalian, China). Specific forward and reverse primers of all the identified (49) *VvIQD* genes were designed using Beacan Designer 7.9 (Table S1). The RT-PCR analysis of all *VvIQD* genes using the cDNA was performed following the guidelines as described in previous studies [28], and actin genes were used as a reference for qRT- PCR [29]. CT- values were used to calculate the relative fold-change. In brief, the real-time PCR amplification reactions were performed on an ABI 7500 Real Time PCR System (Applied Biosystems, USA) using SYBR Green (Vazyme, Nanjing, China) with three replicates. The amplification parameters were: denaturation at 95 °C for 10 min, 40 cycles of denaturation at 95 °C for 15 s, annealing at 60 °C for 15 s, and extension at 72 °C for 15 s.

Previously published RNA-sequencing data from various organs was downloaded with the accessions GSE36128 (different tissues of grape) and GSE98932 (different ripening stages of grapes) [30]. Additionally, FKPM-values (Log_2_) were used to calculate the fold-change in the gene transcriptional levels, and heat maps were illustrated using the RStudio (Version 3.6.1) (R program).

### 2.8. Subcellular Localization and Protein-Protein Interaction Analysis

Epidermal cells of *Nicotiana benthamiana* leaves were transiently transformed with 35S:VvIQDs:GFP (*VvIQD22*, *VvIQD23*, *VvIQD38*, and *VvIQD49*) (A-D). Images were taken under GFP, mCherry, blight field, and merged. The full-length cDNAs of nYFP-*VvIQD49* and cYFP-*VvCaM2* were amplified using the primer pairs provided in the Supplementary Table S1. *VvIQD49* and *VvCaM2* were inserted into the p2YN-35S and p2YC-35S, which contain DNA encoding the N or C-terminal regions of YFP (YFPN or YFPC). After sequence confirmation, the three fusion constructs and the control vector (p2YN-35S and p2YC-35S) were transformed into *A. tumefaciens* strain EHA105 by heat shock. The abaxial surfaces of 5-week-old *N. benthamiana* leaves were transiently co-transformed using an *A. tumefaciens* strain EHA105 infection method with different combinations of these constructs as described by Walter, et al. [31]. YFP-dependent fluorescence was detected 48 h after transfection using a confocal laser-scanning microscope (Zeiss LSM 780, Germany).

### 2.9. Statistical Analysis

The data of RT-PCR were subjected to three-way analysis of variance (ANOVA) for each sample, and for significance, Tukey HSD test was performed with the help of SPSS (Version 16.0, Chicago, USA) at *p* < 0.05 level of significance.

## 3. Results

### 3.1. Identification, Biochemical Properties, and Gene Structure of VvIQDs

**Figure 1:**
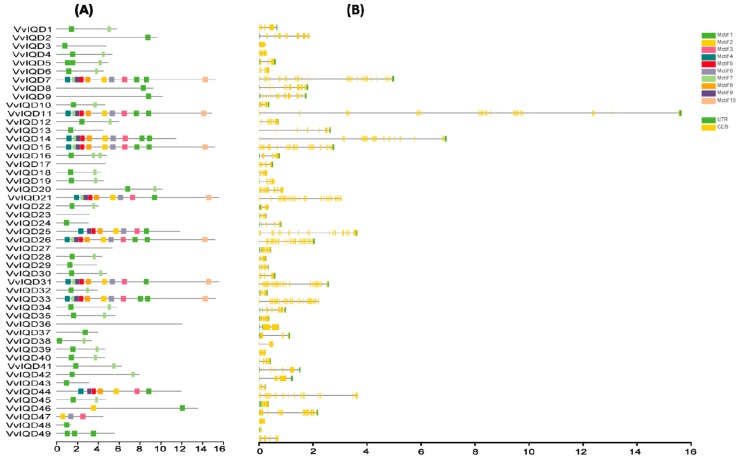
Motif structure of *VvIQDs* and gene structure analysis of IQD members in grapevine (**A**) and (**B**). The coding sequences (CDS), untranslated regions (UTR).

In total 49 putative *VvIQD* genes were identified from the *V. vinifera* genome and were denominated as *VvIQD1* to *VvIQD49* depending on their phylogenetic position relative to their associated orthologous gene pair of *Arabidopsis* (Table S2). Biochemical analysis demonstrated that the cDNA length ranged from 552 bp (*VvIQD48*) to 5042 (*VvIQD7*) with an average cDNA length of 2502.51 bp. Protein length varied from 137 aa (*VvIQD48*) to 1558 aa (*VvIQD21*) with an average protein length of 732.76 aa. The theoretical isoelectric point (pI) and molecular weight (MW) ranged from 4.72 (*VvIQD3*) to 11.02 (*VvIQD49*) and from 34.31822 kDa (*VvIQD24*) to 177.18972 kDa (*VvIQD31)*. The prediction of exon numbers varied from 2 (*VvIQD3* and *VvIQD48*) to 39 (*VvIQD11*, *VvIQD21*, *VvIQD26*, and *VvIQD31*). The sequence features of VvIQDs were analyzed by conserved motifs using the MEME program (Figure 1A). Results revealed 10 conserved motifs (motifs 1–10) with variation in their composition on each *IQD* gene. Motifs 1 and 7 were the most common among all of the members. We also obtained the LOGOS of 10 conserved motifs for IQD, and their distribution is presented in Figure S1. Gene structure organization analysis was performed based on untranslated regions (UTRs) and coding sequences (CDS) of *VvIQD* using TBTools. The *VvIQD* members are highly conserved and exhibited little similarity within subgroups (Figure 1B). Our findings suggest variability between features of the *VvIQD* gene family.

### 3.2. Chromosomal Distribution, Syntenic Relationship, and Divergence Analysis

**Figure 2:**
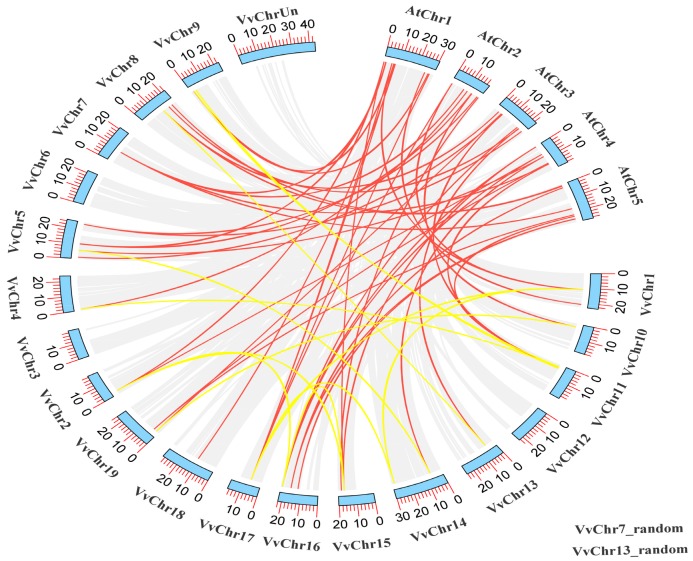
The collinear correlation of *VvIQDs* between grapevines and *Arabidopsis* (indicated with red colored lines) and within grapevine (indicated with yellow colored lines). The two random genes in grapevine genes are also given at the bottom.

To investigate the orientation of the 49 identified *VvIQD* at different chromosomal locations, TBtools software was used to identify the chromosomal positions. The number of *VvIQDs* on the 19 different chromosomes (chr1–chr19) ranged from 1–5 genes per chromosome (Figure 2). The various chromosomal locations had different numbers of *VvIQD* genes, for instance chr5 presented the most *VvIQD* genes (*VvIQD8*–*VvIQD12*), followed by chr8 (*VvIQD16*–*VvIQD19*), chr9 (*VvIQD20*–*VvIQD23*), and chr16 (*VvIQD37*–*VvIQD40*) each with four genes, while chr6 (*VvIQD13*), chr12 (*VvIQD29*), chr17 (*VvIQD41*), and chr18 (*VvIQD42*) had only one gene (Figure 2). No gene was allocated to chr3 location, yet three genes (*VvIQD47*–*VvIQD49*) were allocated to unknown chromosomal (chrUn) locations, indicating that VvIQDs are randomly distributed to different chromosomal locations. The collinear relationship between *V. vinifera* (*VvIQD*) and *Arabidopsis* (*AtIQD*) genes (indicated with red lines), and within the *V. vinifera* genome (indicated with yellow lines), suggest high conservation (Figure 2).

The selective pressure of *VvIQD* paralogous gene pairs and their duplication types were studied by computing the synonymous (*Ks*) and non-synonymous (*Ka*) substitution rate. All of the *IQD* orthologous and paralogous gene pairs exhibited <1.00 (*Ka*/*Ks*) ratio, indicating purifying selection (Table S3).

### 3.3. Phylogenetic Relationship of VvIQD Genes with Various Other Species

**Figure 3:**
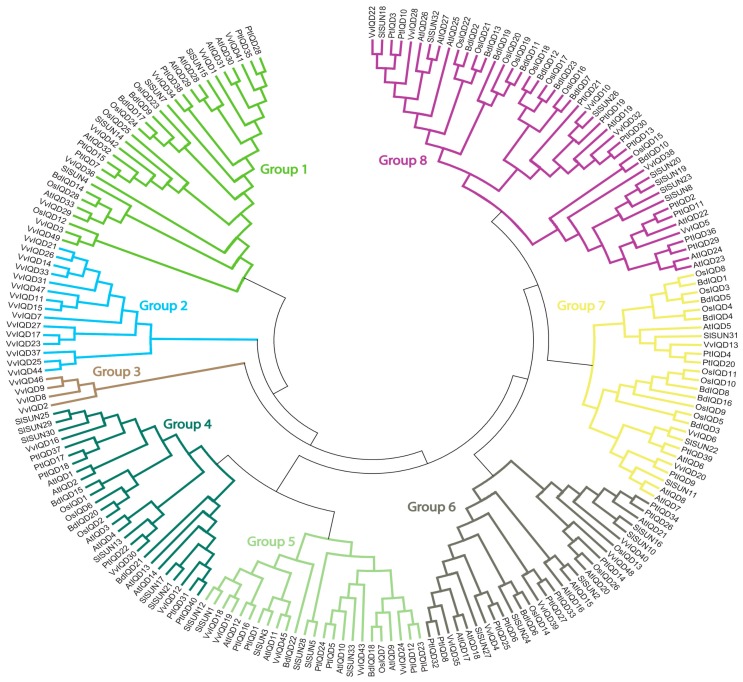
Phylogenetic relationships of *V. vinifera* VvIQD genes with *A. thalinana*, *P. patens*, *O. sativa*, *S. lycopersicum*, and *B. distachyon*. The phylogenetic tree was drawn by using the maximum likelihood (ML) method with 1000 bootstrap replicates in MEGA (Version 7.0).

A phylogenetic tree was constructed with six species, including *Vitis vinfera*, *A. thaliana*, *P. patens*, *O. sativa*, *B. distachyon*, and *S. lycopersicum* to understand the evolutionary relationship of *VvIQD* genes (Figure 3). The resulting phylogenetic tree was sub-classified into eight distinct subgroups. Subgroup 2 had the most IQD proteins (15), followed by subgroup 1 (8) and subgroup 8 (6). Subgroups 5 and 6 contained six *IQD* genes. Subgroups 4 and 7 had the fewest *IQD* genes each with three and two genes. These results suggest that subgroup2 shows large conservation and might share similar functions to its abundance.

### 3.4. Co-regulatory Network Transcriptional Profiling and qRT-PCR Validation

The transcriptional level of 49 *IQD* genes across 54 different tissues was performed, and the full names details and names are given in Table S4. The RNA-seq data was retrieved from NCBI (SRA database) to study the tissue-specific response in *V. vinifera*. A heatmap was constructed on the FPKM-based (Log_2_) values of the 49 *VvIQD* genes to represent tissue-specific expressional discrepancies (Figure S2). Four genes (*VvIQD8*, *VvIQD12*, *VvIQD16*, and *VvIQD46*) showed higher expression in all tissues, indicating their involvement in tissue formation and development. Some genes (*e.g., VvIQD2*, *VvIQD7*, *VvIQD9*, *VvIQD15*, *VvIQD21*. *VvIQD25*, *VvIQD30*, and *VvIQD44*) were moderately expressed in all of the selected tissues, indicating a tissue-specific response in grapevine. The remaining genes responded in any of the tissues, while *VvIQD11*, *VvIQD38*, *VvIQD47*, and *VvIQD48* had low expression abundance compared to the other *VvIQD* proteins, intimating that their participation in tissue or organ development was limited in grapevine (Figure S2).

In addition, we mined RNA-seq data on grape berry development at different time points to understand the role of the VvIQD proteins in the berry ripening process (Figure S3). The *VvIQD8*, *VvIQD36*, *VvIQD37*, and *VvIQD42* genes showed moderate to high transcriptional activity at the early stages of fruit set and development, and were then down-regulated as the grape berry matured (Figure S3). On the contrary, *VvIQD12* and *VvIQD16* showed moderate expression right from fruit stage, then expression was gradually increased with different time points of berry samples (Figure S3). The rest of the *VvIQD* showed moderate, weak, or no expression during berry development, indicating that only a few *VvIQD* genes are involved in ripening process of grape berries.

**Figure 4:**
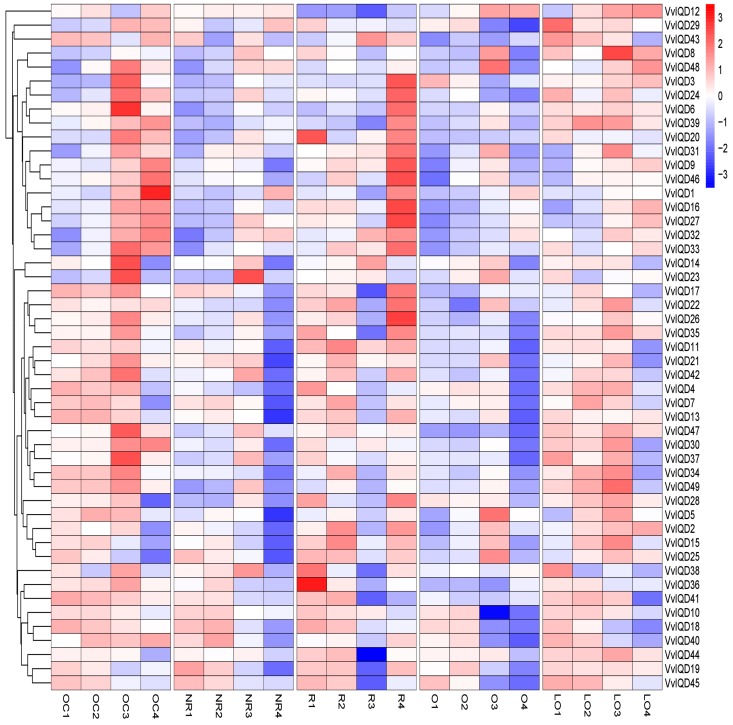
Expression profiles of the *VvIQDs* genes in different grapevine cultivars with distinct shape at different developmental stages. Where OC shows oblate circle; NR, nearly round; R, round; O, oval; and LO, long oval. The numbering (1–4) of each shape indicates *V. vinifera* different developmental stages (i.e., 20daf, 50daf, 80daf, and 110daf).

The IQD proteins are known to regulate shape of harvestable organs in many plants [32]. To validate the role of IQD proteins in grape berry shape development, the expression level of all of the 49 *VvIQD* genes was quantified in five different grapevine cultivars with distinct shape, including oblate circle (OC), nearly round (NR), round (R), oval (O), and long oval (LO) at four different development stages of grapevine development (Figure 4). Moreover, PCA analysis was carried out to check the variability in the relative gene expression level of VvIQDs in grape berries of different shapes during their development. Our findings showed greater variability, exhibiting 70.97% variation in dim1 and 10.45% variation in dim2, with 81.43% cumulative inertia in the first two (dim1 and dim2) axes (Table S5). All the vectors showed positive loadings in both axes (dim1 and dim2), while higher positive loading was recorded in LO2 (6.83), followed by O2 (6.78), whereas lowest loading was found in R1 (0.78) (Figure S4a; Table S5). However, VvIQD genes showed both positive and negative loadings in both dim1 and dim2 (Figure S4a and Table S5). Higher positive loadings were found in VvIQD37 (21.35), VvIQD8 (10.57), and VvIQD12 (5.97), while higher negative loadings were recorded in VvIQD (−2.05), followed by VvIQD20 (−1.99) and VvIQD34 (−1.94) in dim1 and dim2 of the PCA. In addition, correlation analysis also depicted positive correlation between all shapes during grape berry development. Highest positive correlation was found in LO2 (R^2^ = 0.97), followed by O2 (0.96) and LO3 (0.94), while lowest was observed in R1 (0.11) (Figure S4b and Table S5).

### 3.5. Gene Ontology (GO), Kyoto Encyclopedia of Gene and Genome (KEGG) and Promoter Analysis

GO analysis was performed by using the orthologous *A*. *thaliana AtIQD* gene pairs to functionally annotate IQD proteins in three main GO terms. The GO results proposed that IQD proteins were functioning in three main GO classifications, including biological process (40), molecular function (28), and cellular component (15) (Figure S5). In biological processes, ‘vesicle-mediated transport’, ‘cytokinesis’, ‘cell division’, ‘cell cycle’, ‘movement of cell or subcellular component’, and ‘cellular component morphogenesis’ were the most enriched GO terms. Similarly, ‘drug binding’, ‘adenyl ribonucleotide binding’, ‘ATP binding’, ‘adenyl nucleotide binding’, and ‘purine ribonucleoside triphosphate binding’ were the enriched GO terms of molecular function. Other GO terms, such as, ‘myosin complex’, ‘actin cytoskeleton’, ‘cytoskeletal part’, and ‘cytoskeleton’ were the most common GO terms in the cellular component. The KEGG enrichment analysis showed potential involvement of IQD proteins in “brite hierarchies” followed by protein families: genetic information processing, and “cytoskeleton proteins” pathways (Figure S6).

The promoter analysis of the 49 *VvIQD* genes demonstrated their involvement in hormonal signaling (i.e., auxin, SA, MeJA, ABA, and GA-responsive), defense and stress signaling (i.e., drought, cold, and salt), and flavonoid gene regulation (Figure S7).

### 3.6. Subcellular Prediction Analysis of IQD Genes

**Figure 5:**
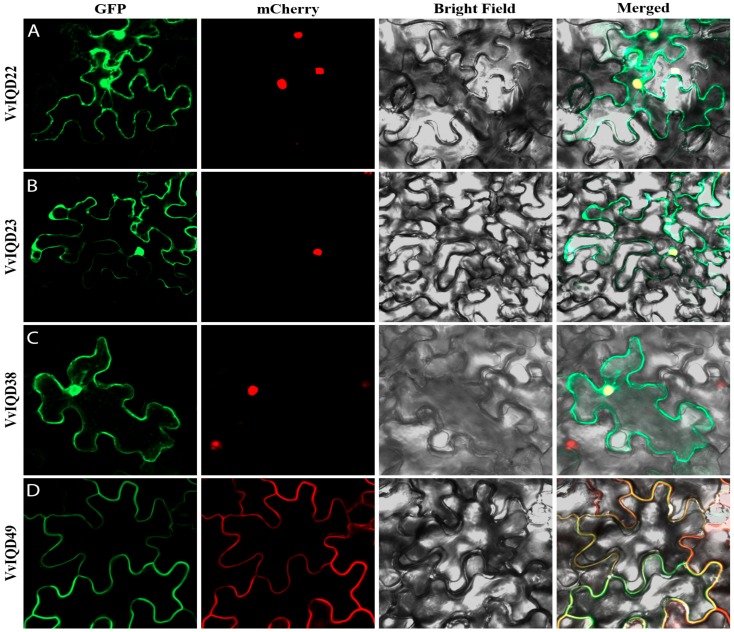
Subcellular localization of *VvIQD22* (**A**), *VvIQD23* (**B**), *VvIQD38* (**C**), and *VvIQD49* (**D**) in nucleus and membrane, using the *N. benthamiana* epidermal cells.

To validate the subcellular localization of certain *IQD* genes, such as *VvIQD22*, *VvIQD23*, *VvIQD38*, and *VvIQD49*, they were transiently transformed in *N. benthamiana* epidermal cells; a construct of *35S-VvIQD:GFP* and *35S-GFP* as a positive vector was used (Figure 5A–D). The GFP-*VvIQD22,* GFP-*VvIQD23,* and GFP-*VvIQD38* fusion proteins were in the nucleus, while GFP-*VvIQD49* was in the membrane of the *N. benthamiana* epidermal cells. The GFP proteins with *VvIQD*s were dispersed throughout the cell.

### 3.7. Protein Interaction Analysis VvIQD with CAM2

**Figure 6:**
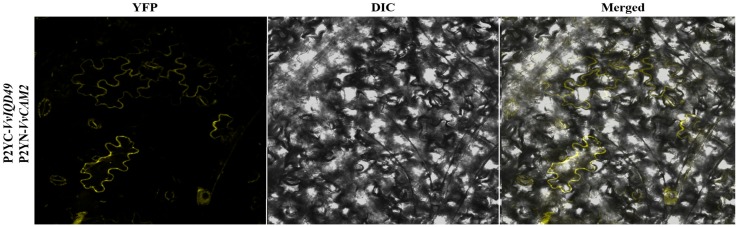
BiFC interaction analysis indicating *VvIQD49* interaction with *VvCaM2* by using the *N. benthamiana* epidermal cells.

The subcellular prediction analysis prompted us to carry out VvIQD protein interaction analysis with VvCaM2 using BiFC assay, to study the protein-to-protein interaction with subcellular resolution. Previous research on *Arabidopsis* and maize demonstrated that IQD proteins can interact with CaM2. The co-expression of nYFP-*IQD49* with cYFP-*CaM2* in *N. benthamiana* system resulted in BiFC signals in the plasma membrane (Figure 6), which is consistent with subcellular prediction of *VvIQD49* (Figure 5D).

## 4. Discussion

Plant-specific *IQD* gene family is ubiquitous in photosynthetic organisms and is known to regulate vital biological processes in plants. Based on the occurrence of one *IQD*-like gene in *P. patens* and nine in *Pinus* sp., *IQD* gene family is believed to originated prior to bryophyte and/ or vascular plants divergence (i.e., 450–700 MYA), but before the divergence of gymnosperms and angiosperms ~300 MYA [33,34]. The comprehensive genome-wide analysis of *IQDs* has been extensively carried out in many model and non-model plants, such as *Arabidopsis*, rice [33], maize [35], soybean [36], poplar [37], and tomato [38]. A precise and systemic characterization of *IQD* gene family in grapevine has not yet been studied in detail. Therefore, extensive genome-wide analysis, including: identification, phylogenetic analysis, motifs and gene structure analysis, GO and KEGG enrichment analysis, subcellular prediction, and protein–protein interaction of VvIQD with VvCaM2 were analyzed in grapevine.

In this study, 49 IQD encoding proteins were identified from the grapevine genome and denominated as *VvIQD1-VvIQD49*, more than previous studies on tomato (34), *Arabidopsis* (33), maize (26), and poplar (40). The grapevine genome (~500 Mbp) with 30,434 protein-coding genes (PCGs) in total (at least five exons on each gene) is larger than poplar (483 Mbp), rice (430 Mbp), and *Arabidopsis* (~130 Mbp). The *Arabidopsis* lineage has experienced two genome duplications (α and β) after the divergence, but grapevine genome has not experienced genome duplication recently, so the identification of ancestral traits and properties of the genomic organization may be possible [34].

Motif analysis in grapevine IQD family showed that the majority of VvIQD proteins had similar motif distribution. Motifs 1 and 7 are the most common, followed by motifs 2 and 4, which indicated that *VvIQD* genes have intricate functionality in grapevine. The motif distribution in different *VvIQD* genes may impart their functional diversity. The selection pressure on gene pairs (i.e., positive, purifying, and neutral) intimates the vital information related to rate of divergence [29]. The duplicated *VvIQD* gene pairs exhibited *Ka*/*Ks* ratio <1.00, specifying a positive selection; similar findings have previously been reported for grapevine [29].

To comprehend the possible involvement of the *VvIQD* proteins in grapevine growth and development, transcription levels of 49 *VvIQD* genes across 54 different tissues and organs were detected from transcriptomic data. Data revealed apparent functional diversity; *VvIQD8*, *VvIQD12*, *VvIQD16*, and *VvIQD46* were all more highly expressed in all 54 tissues. The low expression level of the other *VvIQD* genes suggests that these genes work synergistically with corresponding interacting proteins during tissue or organ development. Furthermore, we used the RNA-seq data to validate the role of *VvIQDs* in the berry-ripening process, which suggested two (*VvIQD12* and *VvIQD16*) marker genes, with increased expression during grape berry ripening. Many studies have demonstrated that *IQD* gene can alter fruit shape, such as *IQD12/SUN* locus, which can alter the cell shape and produce elongated tomato fruits [39]. To understand the critical role of *VvIQD* genes in grape berry shape development, qRT-PCR analysis was performed to detect the gene-expression in fruits of different shapes. Results pointed to four candidate genes involved in shape development: *VvIQD3* in oblate circle and round, *VvIQD4* in oblate circle and long oval, *VvIQD6* in oblate circle, and *VvIQD18* in nearly round. Further functional characterization of any of these candidate genes can validate their role in shape development.

The GO-based enrichment analysis demonstrated that some *VvIQD* genes are involved in cell division and cellular component morphogenesis, playing an important role in altering cell shape. KEGG analysis also revealed the role of *VvIQD* genes in the cytoskeleton protein pathway, which controls the position of the nucleus [40], which is needed for the shape formation. Finally, we validated the subcellular localization of GFP-fused *VvIQD22, VvIQD23, VvIQD29, VvIQD38,* and *VvIQD49* genes using *N. benthamiana* epidermal cells. The GFP-tagged signals of *VvIQD22, VvIQD23, VvIQD38,* and *VvIQD49* genes were concerted in the nucleus and plasma membrane. The localization and functions of selected genes from nucleus and plasma membrane is in line with the previous findings on *Arabidopsis* [33]. The protein–protein interaction analysis between *VvIQD49* and *VvCaM2* was verified by BiFC analysis in the plasma membrane of *N. benthamiana*, which is consistent with the results of a similar study in *Arabidopsis* [8].

## 5. Conclusions

In this study, extensive genome-wide analysis of *IQD* gene family was carried out using the advanced bioinformatic analyses, which identified 49 *VvIQD* genes in the grapevine genome. Motifs 1 and 7 were the most common of all the genes, and Mapchart assisted in locating *VvIQD* genes at 19 different chromosomal locations. GO and KEGG analysis demonstrated that *VvIQD* is involved in cell division, cellular component morphogenesis, and cytoskeleton protein, which are considered to be involved in altering cell shape, thus regulating fruit shape. The subcellular prediction of *VvIQD22, VvIQD23, VvIQD38,* and *VvIQD49* genes validated their localization in the nucleus and plasma membrane. This study also provides a list of candidate genes that can regulate different grapevine shapes. The overexpression of these genes may provide useful information about the regulation of fruit shape in grapevine.

## Supplementary Materials

The following are available online at https://www.mdpi.com/2073-4425/11/2/235/s1, Figure S1. LOGOS representation of the conserved motifs (1–10). Figure S2. Transcriptional profiling of 49 *VvIQDs* in grapevine berries during different developmental stages. Figure S3. Transcriptional profiling of 49 *VvIQDs* in organ and/ or tissue development of grapevine. Figure S4. The principal component analysis (A and B) of various organs used in our study. Figure S5. Geno ontology (GO) based enrichment analysis of *VvIQDs* in the grapevine genome. Figure S6. Kyoto encyclopedia of gene and genome (KEGG) analysis of *VvIQDs* in the grapevine genome. Figure S7. Promoter analysis of *VvIQDs* in the grapevine genome. Table S1. List of the primers used for the qRT-PCR analysis. Table S2. Biochemical properties, including protein length, chromosomal position, isoelectric point (pI), molecular weight (MW), and subcellular prediction of *VvIQD* genes. Table S3. *Ka/Ks* values of the homolog and ortholog gene pairs compared with *Arabidopsis*. Table S4. The transcriptional profile of 54 different organs. Table S5. The Principal Component Analysis of RT-PCR data.
