# Supplementary figures and images for: Comprehensive Sequence Analysis of IQD Gene Family and their Expression Profiling in Grapevine (Vitis vinifera)
Source: Genes (Basel). 2020 Feb 24;11(2):235. doi: 10.3390/genes11020235 (PMC7073947; doi:10.3390/genes11020235)

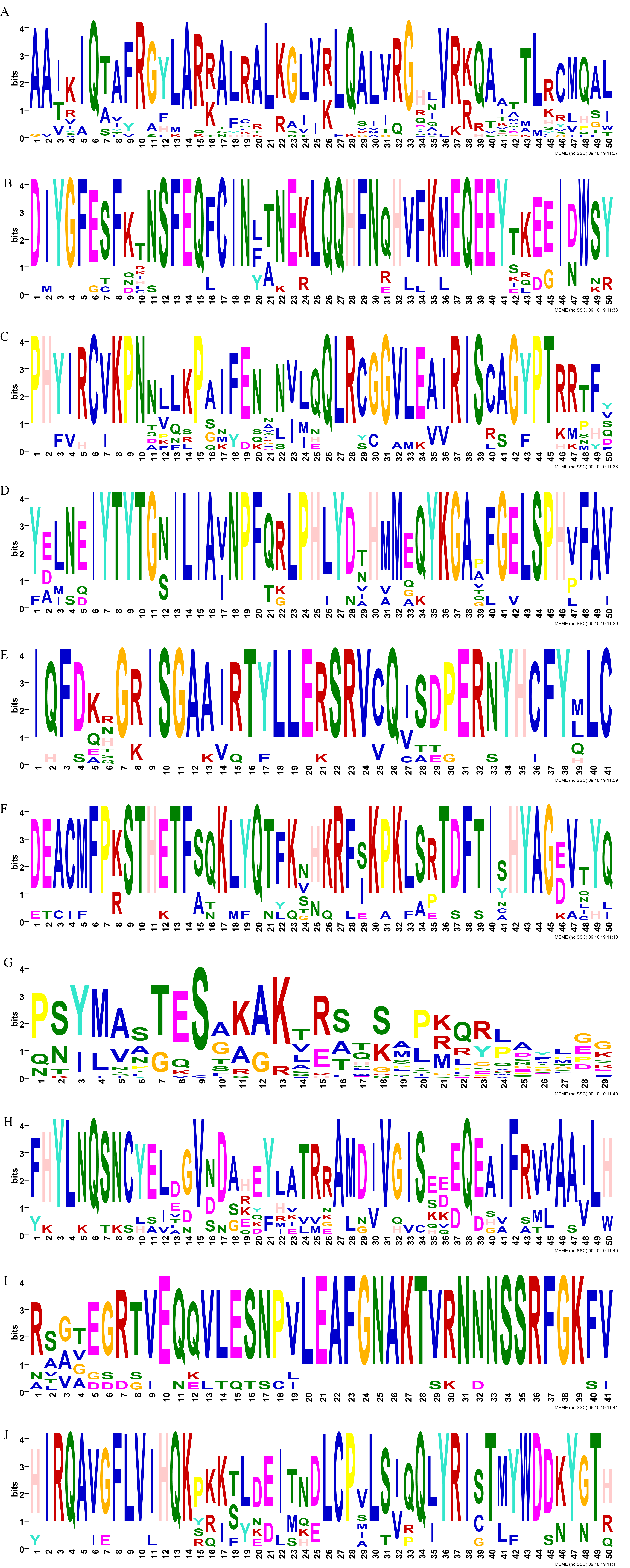

Supplement: Supplementary file 1 [file genes-11-00235-s001.zip › supplementary/Figure S1.tif]

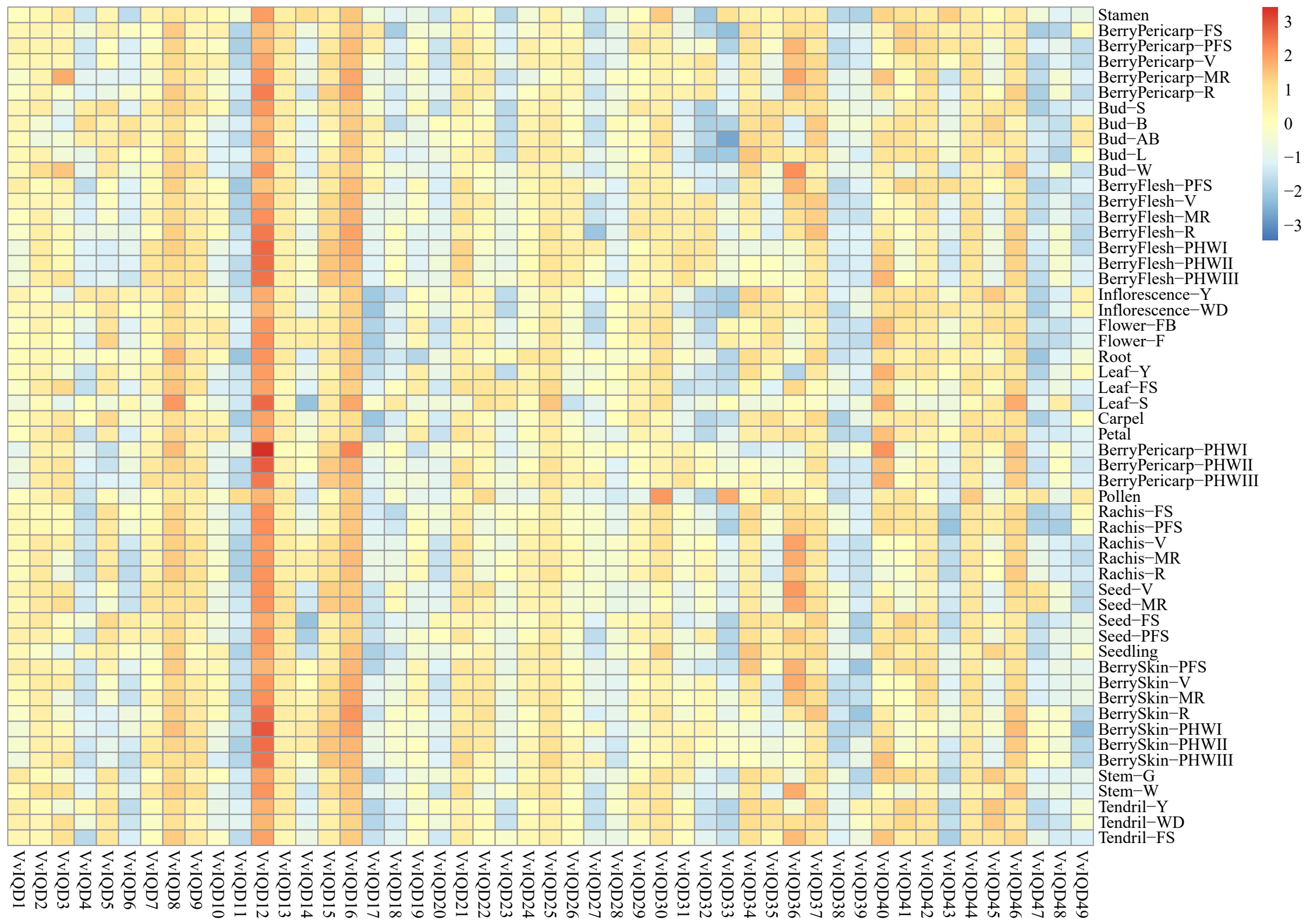

Supplement: Supplementary file 1 [file genes-11-00235-s001.zip › supplementary/Figure S3.pdf]

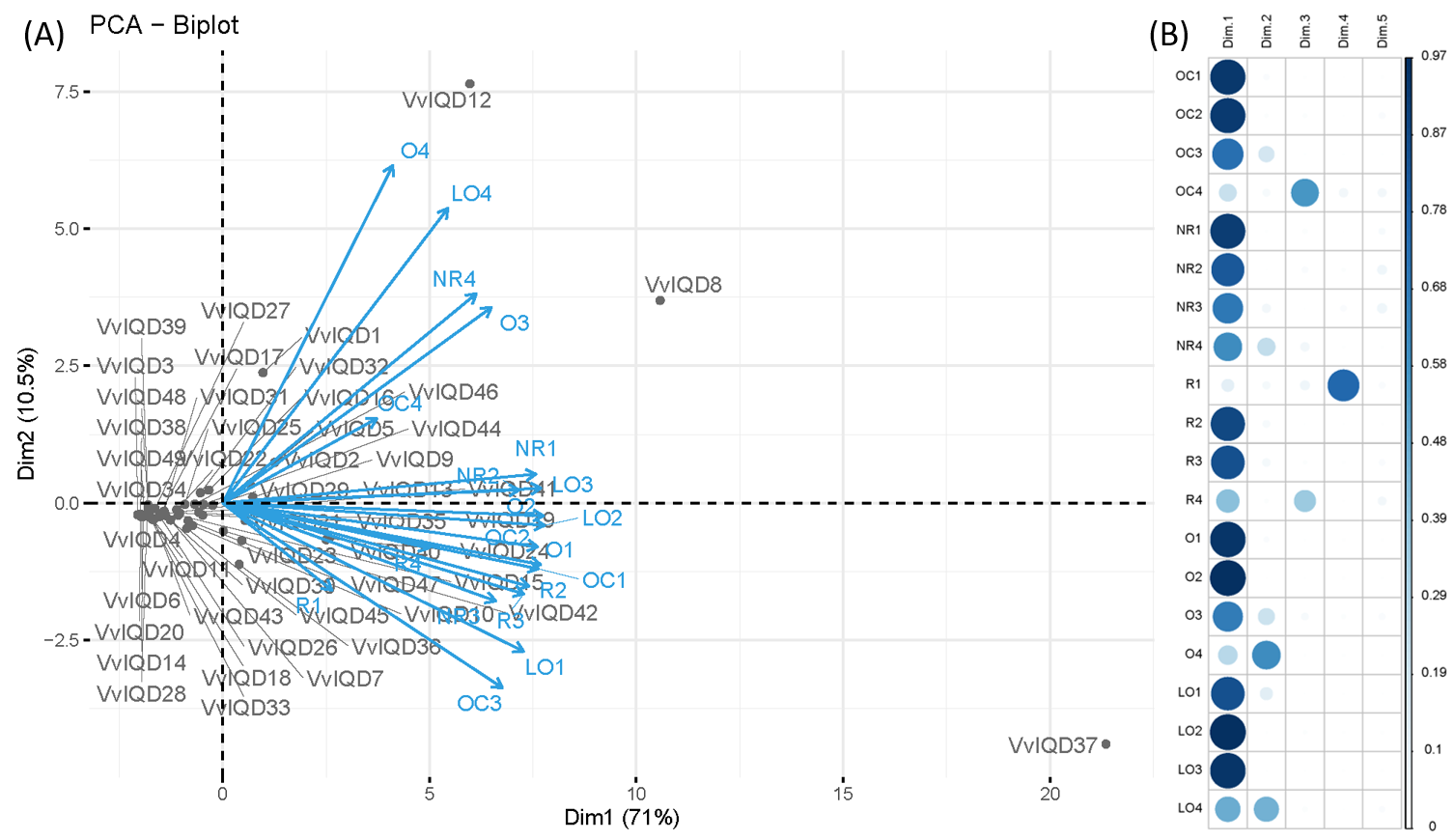

Supplement: Supplementary file 1 [file genes-11-00235-s001.zip › supplementary/Figure S4.tif]

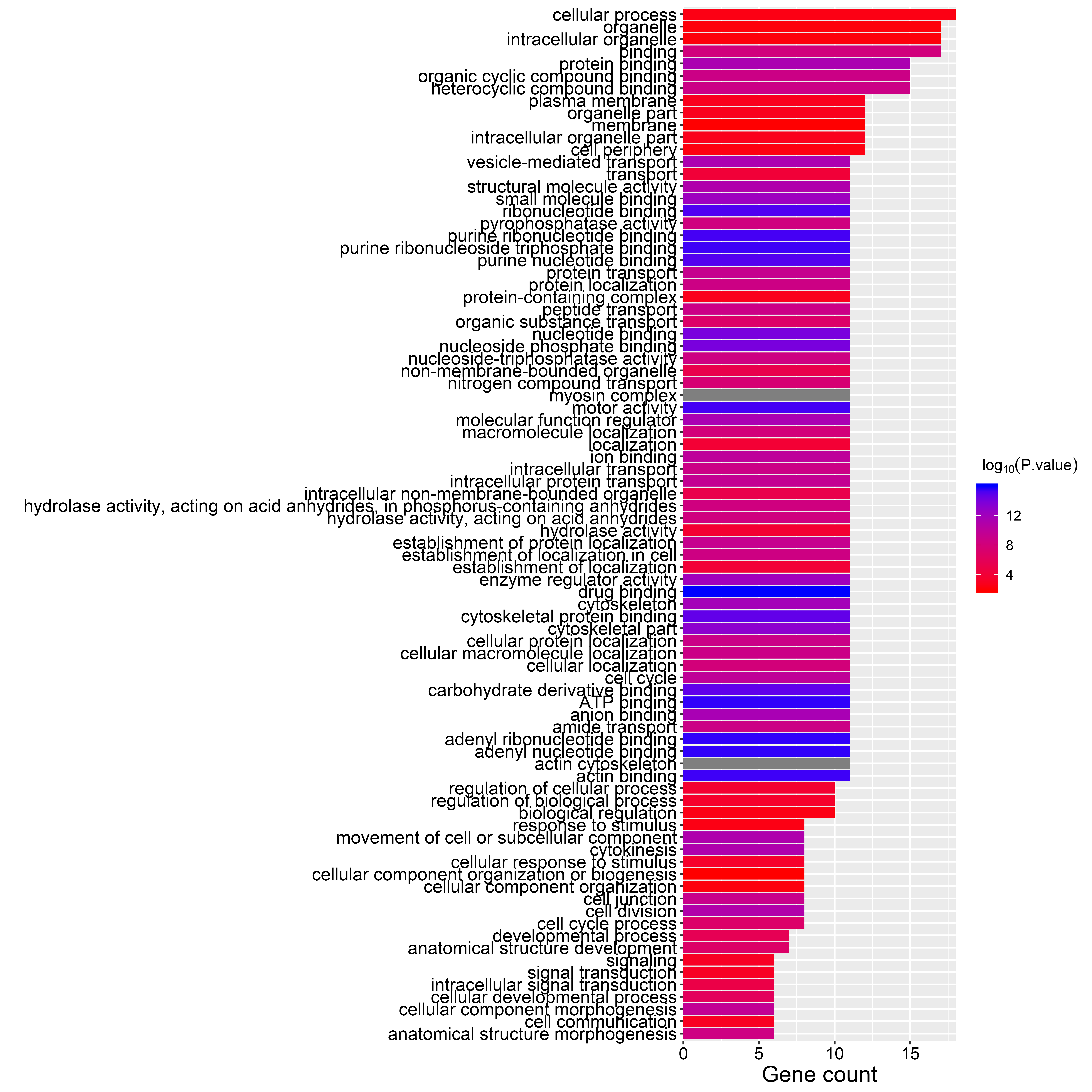

Supplement: Supplementary file 1 [file genes-11-00235-s001.zip › supplementary/Figure S5.png]

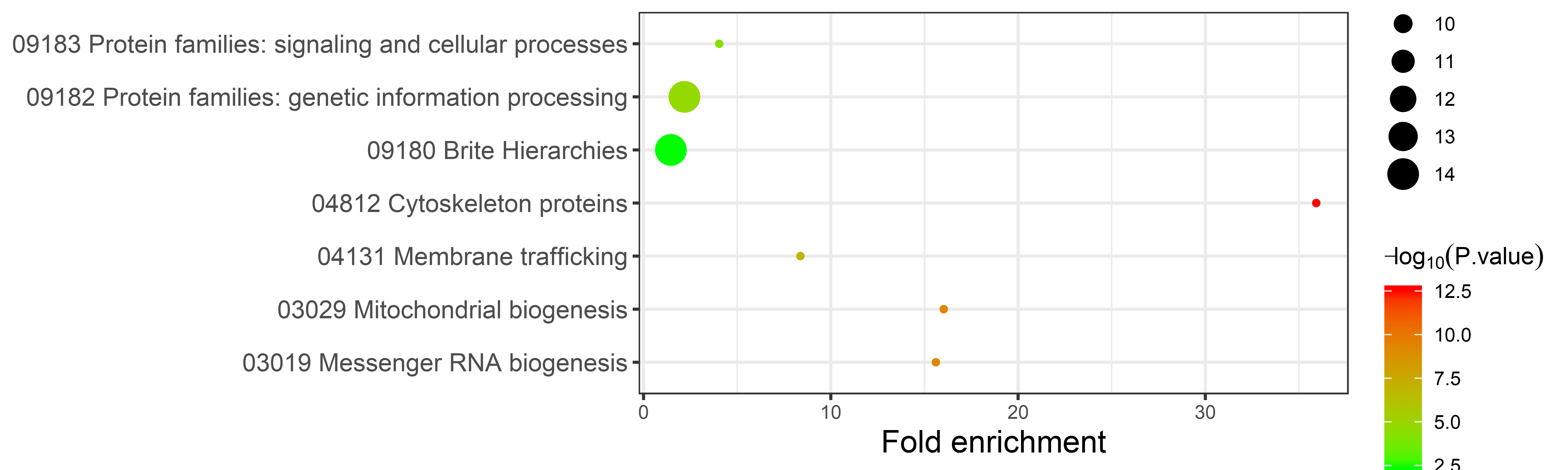

Supplement: Supplementary file 1 [file genes-11-00235-s001.zip › supplementary/Figure S6.png]

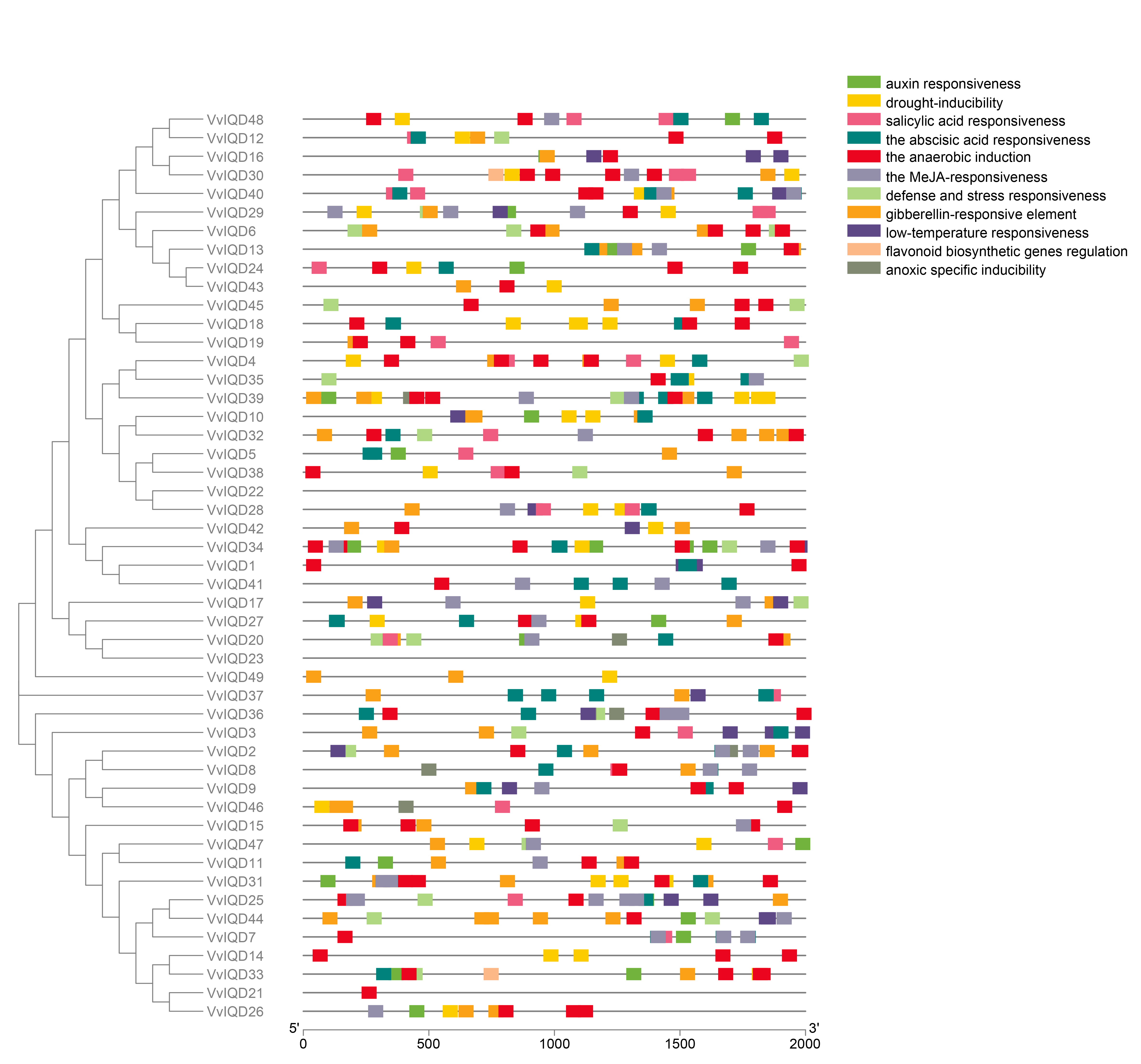

Supplement: Supplementary file 1 [file genes-11-00235-s001.zip › supplementary/Figure S7.tif]
